# Comprehensive Immunoprofiles of Renal Cell Carcinoma Subtypes

**DOI:** 10.3390/cancers12030602

**Published:** 2020-03-05

**Authors:** Moonsik Kim, Jin Woo Joo, Seok Joo Lee, Yoon Ah Cho, Cheol Keun Park, Nam Hoon Cho

**Affiliations:** 1 Deptartment of Pathology, Yonsei University College of Medicine, Seoul 03722, Korea; TEIROA83@yuhs.ac (M.K.); DARKJOOGGA@yuhs.ac (J.W.J.); VIPERINE@yuhs.ac (S.J.L.); 2 Deptartment of Pathology and Translational Genomics, Samsung Medical Center, Sungkyunkwan University School of Medicine, Seoul 06351, Korea; PURPLEICE21@yuhs.ac; 3 Deptartment of Pathology, Armed Forces Capital Hospital, Seongnam 13574, Korea; pck0111@naver.com; 4 Brain Korea 21 PLUS Project for Medical Science, Yonsei University, Seoul 03722, Korea

**Keywords:** renal cell carcinoma, immunohistochemistry, subtyping, diagnosis, algorithm

## Abstract

In recent years, renal epithelial tumors have been among the fastest reclassifying tumors, requiring updates to the tumor classification system. Nonetheless, immunohistochemistry (IHC) remains the most widely used tool for renal epithelial tumors. In this proposal, we aimed to create the most efficient IHC panel for categorizing the diverse subtypes of renal tumors, and to find out more specific immunohistochemical results in each subtype or each antibody. A total of 214 renal tumors were analyzed using 10 possible IHC markers to differentiate subtypes, including three major renal cell carcinoma (RCC) subtypes, clear-cell type (50 cases), papillary type (50 cases), and chromophobe type (20 cases), and minor subtypes (MiT RCC, 13 cases; collecting duct carcinoma, 5 cases; and oncocytoma, 10 cases). A triple immunomarker (cytokeratin 7 (CK7)-carbonic anhydrase IX (CAIX)- alpha-methylacyl-CoA racemase (AMACR)) panel is useful in particular high-grade clear-cell tumors. If IHC remains ambiguous, the use of an adjunctive panel can be suggested, including CD10, epithelial membrane antigen, cathepsin K, c-kit, hepatocyte nuclear factor 1-β, and E-cadherin. For an efficient immunohistochemical strategy for subtyping of RCC, we conclude that the CK7-CAIX-AMACR panel is the best primary choice for screening subtyping.

## 1. Introduction 

For cancer diagnosis, immunohistochemistry (IHC) results often serve as valuable diagnostic tools when staining is positive. However, when using IHC to subtype renal cell carcinoma (RCC), both positive and negative staining of key immunomarkers are comparatively important. These RCC subtypes include clear-cell tumors, such as clear-cell carcinoma (ccRCC), chromophobe RCC (ChRCC), clear-cell papillary RCC (CCPRCC), and microphthalmos translocation family RCC (MiT RCC). Alternatively, eosinophilic tumors can be seen in papillary RCC (PRCC), CCRCC, ChRCC, eosinophilic variants, renal oncocytoma (RO), and acquired cystic disease RCC (ACD-RCC) [1,2,3,4,5,6,7].

Ancillary triage of RCC tumors is mandatory except for tumors with very typical histological findings, as overlapping morphology is common and each subtype has a different prognosis. However, IHC results are often ambiguous and can lead to misdiagnosis. Furthermore, routine histological results can frequently overlap between similar subtypes. Therefore, more definitive immunohistochemical panels are required for cardinal subtyping. 

In this study, we revisited the immunohistochemical landscape according to RCC subtyping since the 2012 Vancouver renal tumor classification to propose five new tumors and three emerging new entities, and refine existing subtypes [2]. Of the markers studied, a few easily differentiated into a two-tier system of positive or negative; however, most markers were difficult to evaluate as simply positive or negative, due to focal patch staining that lacked golden cut-off criteria. If not given any scientific or practical guidelines, simply categorizing IHC results into a two-tier system may lead to serious misinterpretations. Therefore, we suggest the application of both estimations of quantitative staining and analysis of marker expression patterns.

Another goal of this study was to create definitive IHC panels for RCC diagnosis, as laboratory trial access to renal tumors is highly variable. Based on our results, we suggest a two-step approach: first with a triple-biomarker panel, and subsequently, with more specific panels. The first triple-biomarker panel was sufficient to subtype nearly 90% of renal tumors. However, new RCC subtypes or those unclassified even by the triple-biomarker panel required subsequent ancillary tools. Although a large number of antibodies could be applied for RCC subtyping, a non-triage approach would lead to both time and financial burdens. Alternatively, we systematically reviewed IHC findings from variable subtypes of RCC to create a comprehensive algorithm of IHC for solid subtyping. 

## 2. Results 

**Figure 1:**
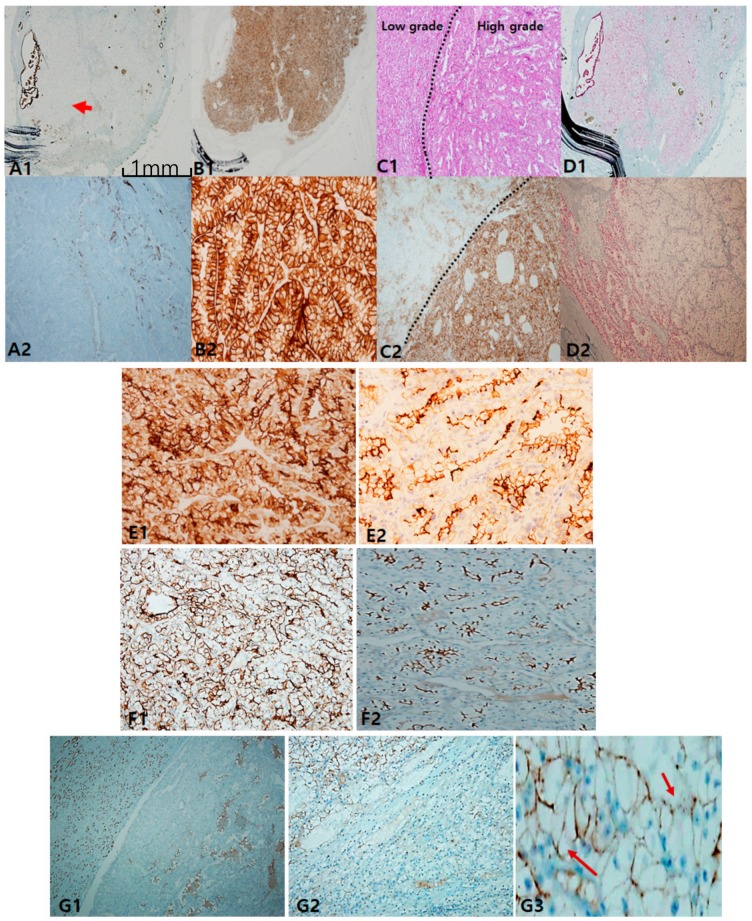
Classic immunopanels for clear-cell renal cell carcinoma (CCRCC). (**A1**,**A2**) cytokeratin 7 labeling is mostly negative but exceptionally positive in macrocystic tubules (arrow). (**B1**,**B2**) Carbonic anhydrase IX (CAIX) is always positive as a box pattern, which is a pathognomonic sign. (**C1**,**C2**) CAIX labeling tends to be stronger and more diffuse in higher grades (grade 3–4) than in lower ones (grade 1–2). (**D1**,**D2**) Alpha-methylacyl-CoA racemase is variably positive but less intense than in papillary renal cell carcinoma (PRCC). Adjuvant immunopanels of CCRCC. (**E1**,**E2**) In CCRCC, immunoreactivities of epithelial membrane antigen and CD10 are closely correlated. EMA is more exaggerated (E1) and CD10 expression occurs in a saw-tooth luminal pattern (**E2**). (**F1**,**F2**) EMA (**F1**) labeling recapitulates exaggerated CD10 (**F2**) expression. (**G1–G3**) E-cadherin expression is frequently lost (**G1**,**G2**) or occasionally broken (arrows) (**G3**). Scale bars = 1 mm.

In clear-cell carcinoma, cytokeratin 7 (CK7) was negative or occasionally focal positive, but it was not found to be diffusely positive. The exception was that intense staining could be seen in pseudopapillary and macrocystic spaces (Figure 1A). When intense and diffuse CK7 staining is observed in a clear-cell-predominant tumor, the possibility of CCRCC is less likely. Alternatively, carbonic anhydrase IX (CAIX) was diffusely and intensively stained with a box pattern (surrounding each quadrangle border) in all CCRCC, which can play a role in a pathognomonic marker (Figure 1B). Importantly, CAIX staining was inversely correlated with CK7 staining; thus, the stronger the CAIX staining, the less CK7 is stained. Colocalization of CK7 and CAIX seldom occurred. Alpha-methylacyl-CoA racemase (AMACR) staining was more variable in CCRCC (Figure 1D). In terms of prognostication, tumor cells with worse grades tended to express more CAIX (Figure 1C). Despite less specificity, CD10 can be used to differentiate CCRCC from non-CCRCC, particularly PRCC, according to the staining pattern. The former is characterized by a sawtooth pattern along a scalloped luminal contour (Figure 1E), whereas the latter demonstrates a compressed luminal appearance in PRCC (Figure 1F). Furthermore, we noticed that CD10 inversely correlates with CK7 expression. E-cadherin was frequently lost (Figure 1G1,G2) or occasionally broken (Figure 1G3).

**Figure 2:**
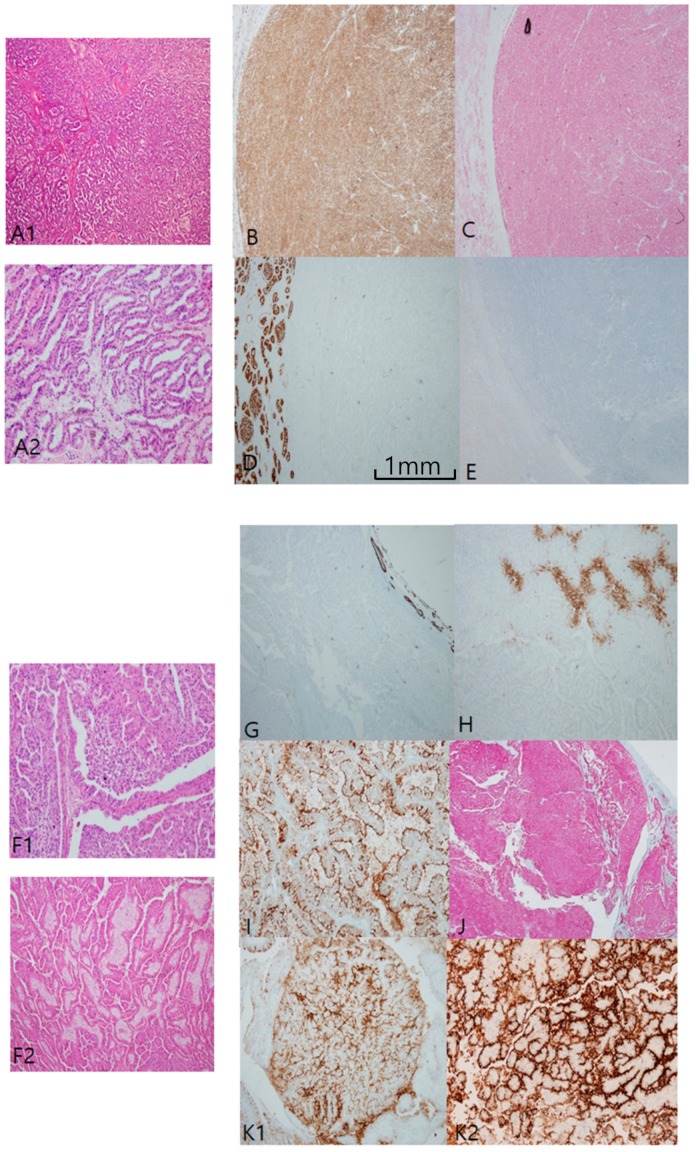
Papillary renal cell carcinoma (PRCC), type I. (**A1**,**A2**) Uniform structure predominantly composed of small slit-like tubules and luminal branched papillae is a characteristic of PRCC, type I. (**B**–**E**) PRCC type I is characteristically cytokeratin 7 (CK7)++ (**B**)/Alpha-methylacyl-CoA racemase (AMACR)++ (**C**)/CD10− (**D**)/carbonic anhydrase IX (CAIX)− (**E**). PRCC, type II. (**F1**,**F2**) PRCC type II differs from type I in larger and more complicated papillae line by taller larger cells. Aggregates of foamy histiocytes in the stroma of papillae are frequently noted. (**G**–**K**) Immunohistochemical (IHC) characteristic of PRCC, type II. PRCC, type II is usually CK7 negative (**G**), CAIX negative (**H**), epithelial membrane antigen+ (**I**) AMACR++ (**J**), and CD10++ (**K1**,**K2**). CD10 is basally distributed with a central sparing clubbing pattern (**K2**). Scale bars = 1 mm.

In papillary RCC type I (Figure 2A)*,* both CK7 (Figure 2B) and AMACR (Figure 2C) were constantly positive, while both CD10 (Figure 2D) and CAIX (Figure 2E) were negative. Those distinctive paired profiles seem to be virtually unique to PRCC type I. Total E-cadherin loss of expression was common in PRCC type I. In contrast to PRCC type I, PRCC type II (Figure 2F) is variably different in immunoprofiles. Generally, neither CK7 (Figure 2G) nor CAIX (Figure 2H) were well stained; in fact, both were often patch-positive, insufficient for positive interpretation. Instead, EMA (Figure 2I), AMACR (Figure 2J), and CD10 (Figure 2K1) were constantly positive. CD10 and EMA were stained along compressed luminal borders between papillary tracts, or a “clubbing sign” in typical papillary fronds (Figure 2K2). It was inversely correlated with CK7 expression. E-cadherin labeling was mostly lost, as in CCRCC. Three fumarate hydratase-deficient RCCs were confirmed by next generation sequencing, histologically compatible with the unique features of meganucleoli with papillary architecture. All three were beyond stage 3, and one case revealed a sarcomatoid appearance with reginal node and distant metastasis. With the exception of a focal positive reaction to paired box gene 8 and AMACR, major conventional markers were nearly negative. Instead, fumarate hydratase showed immunoreactivity loss in tumor cells (Figure S1). 

**Figure 3:**
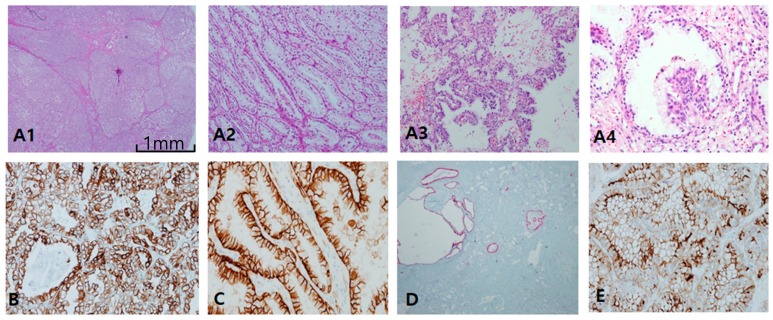
Clear-cell papillary renal cell carcinoma. (**A1**–**A4**) In clear-cell papillary RCC (CCPRCC), histological findings include multilobulated small masses (**A1**) entirely composed of well-differentiated simple tubular structures mimicking distal tubules (**A2**) with intraluminal tufts (**A3**) and short papillary fronds in a glomeruloid pattern (**A4**). (**B**–**D**) Immunohistochemical profiles. Cytokeratin 7 is diffusely and strongly positive (**B**), Carbonic anhydrase IX is positive with cup-shaped pattern, (**C**), Alpha-methylacyl-CoA racemase is generally negative except for macrocystic spaces (**D**), and CD10 as a saw-tooth luminal pattern (**E**). Scale bars = 1 mm.

**Figure 4:**
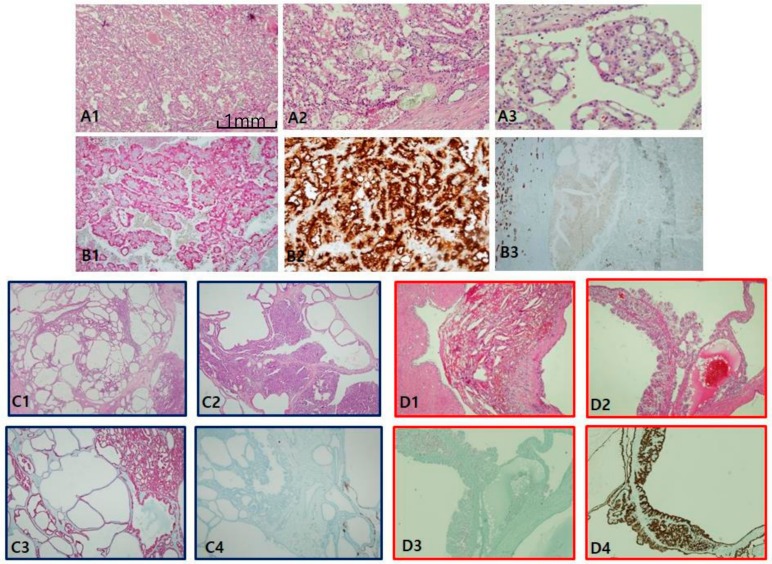
Acquired cystic disease-associated renal cell carcinoma (ACD-RCC). (**A1**–**A3**) In ACD-RCC, characteristic feature includes lace pattern (**A1**) with intratumoral oxalate crystals (**A2**) and coalescent vacuoles (**A3**). (**B1**–**B3**) Immunoprofiles of ACD-RCC. Similar to papillary renal cell carcinoma type II, ACD-RCC demonstrates high alpha-methylacyl-CoA racemase (AMACR; **B1**) and CD10 (**B2**) expression with cytokeratin 7 negativity (**B3**). Immunohistochemistry is needed to differentiate between tubulocystic carcinoma (TCC) and multilocular cystic renal neoplasms. TCC is partly cystic (**C1**) and partly tubular (**C2**), which is characteristically AMACR+ (**C3**) and carbonic anhydrase IX (CAIX)-negative (**C4**) tumor, whereas multilocular cystic RCC shows hemorrhage-filled cyst (**D1**) and heaped-up profiles of clear cells (**D2**), and inverse immunoprofiles to TCC, AMACR-negative (**D3**), and CAIX-positive (**D4**) findings. Scale bars = 1 mm.

Clear-cell papillary RCC (CCPRCC) is found by a couple of pathological clues: well-encapsulated, well-demarcated, and extremely well-differentiated tubular growths with reverse polarity along the luminal juxtaposition, and low International Society of Urological Pathology grade (1/2) and low stage (pT1) (Figure 3A). In contrast to CCRCC, CCPRCC manifested strong and diffuse CK7 staining (Figure 3B). Alternatively, negative or focally positive CK7 in any clear, cell-rich tumor can be against CCPRCC. CAIX is considered a specific cup-shaped pattern with an open luminal aperture (Figure 3C). However, this pattern is not always observed in CCPRCC. Furthermore, cup-shaped CAIX staining can be seen occasionally in CCRCC, particularly in areas of pseudopapillary pattern. AMACR is usually negative but can be focally positive in cystic dilated tubules or papillary fronds (Figure 3D). CD10/EMA staining is quite similar to CCRCC (Figure 3E). EMA and E-cadherin are also positive. Whether associated with end-stage renal disease or not, CCPRCC can develop multilocular cystic manifestations. Immunoprofiles among cystic renal tumors are listed in Table 1. Acquired cystic disease-associated RCC (ACD-RCC) is characterized by multifocal papillary excrescences in the eccentric mural growths of multiple cysts, with lacy sieve-like patterns of obscure cell borders and intratumoral oxalate crystals (Figure 4A). CK7 is generally negative, but can be focally positive along the macrocystic space. CD10 is unanimously positive, while CAIX is always negative. Thus, the IHC portfolio of ACD-RCC is similar to PRCC type II (Figure 4B). A multilocular cystic renal neoplasm of low malignant potential shows strong positivity on CAIX, with no expression of AMACR, whereas tubulocystic carcinoma (TCC) is characteristically reverse to the former, AMACR+ (Figure 4C) and CAIX− (Figure 4D). 

Semiquantitive data of the first triple panel and additional panel in major subtyping of renal tumors are summarized in Table 2 and Table 3, respectively.

In terms of the first triple panels, CCRCC resembles CCPRCC, while cytokeratin 7 (CK7) is useful in both subtypes. Among the distal nephron derivatives such as chromophobe RCC, oncocytoma, collecting duct carcinoma, and tubulocystic carcinoma, CK7 is convenient to screen up. Microphthalmos gene translocation-associated renal cell carcinoma and oncocytoma are immunophobic in triple markers.

**Figure 5:**
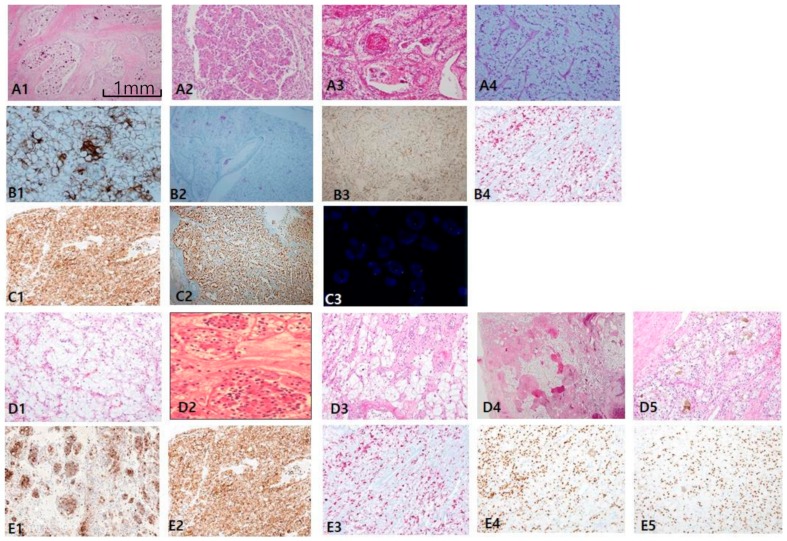
Microphthalmos translocation family RCC (MiT RCC). (**A1**–**A4**) In MiT RCC, characteristic findings include intracystic papillary masses composed of clear cells (**A2**) with psammoma bodies (**A1**) and fibrins (**A3**) and sometimes marked reverse polarity (**A4**). However, these findings are not specific to diagnose MiT RCC by histology alone. (**B**,**C**) When MiT RCC is suspected, adequate immunohistochemical panel should be applied, beginning with cathepsin K staining (**C1**). Conventional markers such as CK7 (not shown), epithelial membrane antigen (**B1**), alpha-methylacyl-CoA racemase (**B2**), and carbonic anhydrase IX (not shown) are generally negative, while HMB45 (**B3**) and melan A (**B4**) are occasionally positive. A more definitive diagnosis can be made with transcription factor E3 (C2) or fluorescent in situ hybridization break-apart signals (**C3**). (**D1**–**D5**) The characteristic findings transcription factor EB (TFEB)-related MiT RCC include nested fluoret sign (**D1**,**D2**), ballooning clear cells (**D3**), stromal hyalinization, fibrinous occlusive blood vessels with extensive peliosis-like changes (**D4**), and melanin pigment deposit in tumor cells (**D5**). (**E1**–**E4**) Immunohistochemical profiles. TFEB (**E1**,**E2**), cathepsin K (**E3**), HMB45 (**E4**), paired box gene 8 (**E5**) are specifically positive. Scale bars = 1 mm.

The most consistent and specific histologic findings of MiT RCC are: (1) frequent psammoma bodies (Figure 5A1), (2) an intracystic mass composed of clear cells with a flocculent ballooning cytoplasm (Figure 5A2), (3) an intraluminal tuft with a basement membrane-like hyalinized core with floral or rosette signs (Figure 5A3), and (4) papillary features composed of hybrid clear and eosinophilic cells, often with reverse polarity (Figure 5A4). MiT RCC, which was confirmed by IHC and fluorescent in situ hybridization (FISH), can be simplified as an immunophobic tendency; CK7 is generally negative to focally patchy (figure not shown), and EMA (Figure 5B1), CAIX (figure not shown), and CD10 (figure not shown) are also negative. AMACR varies from negative to focally positive at intraluminal tufts like a floral (pseudo-rosette) sign (Figure 5B2). However, melanoma-related markers show relatively high expression, such as HMB45 (Figure 5B3) and melan-A (Figure 5B4). Cathepsin K (Figure 5C1) is a more sensitive and specific marker than transcription factor E3 (TFE3; Figure 5C2) for MiT RCC. We identified additional characteristic histologic clues for TFEB-related MiT RCC (TFEB-RCC): a biphasic nested or pseudo-rosette pattern (Figure 5D1,D2), ballooning clear (Figure 5D3) cells with peliosis changes (Figure 5D4), and melanocytic differentiation (Figure 5D5). The most specific clue is positive for transcription factor EB (TFEB; Figure 5E1–2). Cathepsin K is also strongly expressed in TFEB-RCC (Figure 5E3). As shown in the present 14 cases of MiT RCC, cathepsin K was expressed in 85.7% of TFE3+ cases, and was constantly positive in TFEB-RCCs. These results are consistent with recently documented results showing 60% (6/10) positivity in TFE3 and 100% (7/7) in TFEB. HMB45 (Figure 5E4) and PAX8 (Figure 5E5) are also positive in TFEB-RCC. 

**Figure 6:**
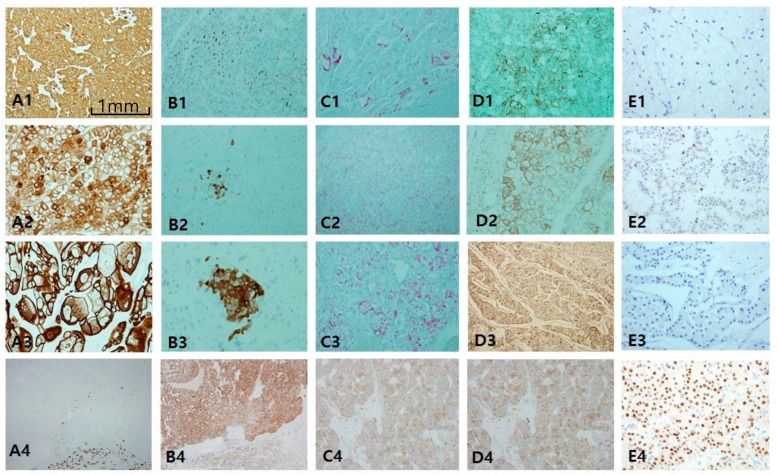
Comparison of chromophobe renal cell carcinoma (ChRCC) with renal oncocytoma (RO). Differential diagnosis between these entities is difficult since they overlap in origin and morphology. (**A1**–**A4**) cytokeratin 7 is highly intense and diffusely stained in ChRCC in contrast to RO showing perfect negativity. (**B1**–**B3**) CD10 is generally negative, but focally patch positive, which is ignored. (**C1**–**C3**) Alpha-methylacyl-CoA racemase is negative or patch positive. Epithelial membrane antigen (**B4**) and E-cadherin (**C4**) are diffusely positive both in ChRCC and RO. (**D1**–**D4**) c-kit staining is basically positive in both entities. (**E1**–**E4**) Hepatocyte nuclear factor-1β (HNF1β) staining is a highly useful immunomarker to rule out the possibility of ChRCC. Scale bars = 1 mm.

**Figure 7:**
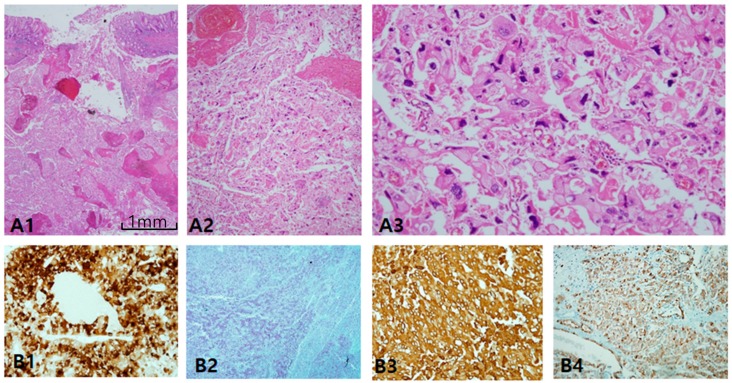
Alteration of immunoprofiles in aggressive chromophobe renal cell carcinoma (ChRCC). (**A1–A3**) Aggressive behavior is uncommon in ChRCC, but few cases show metastasis and even rupture intestine (**A1**). Tumor cells lost the nature of ChRCC, showing sarcomatoid differentiation (**A2**) and marked anaplasia (**A3**) corresponding to International Society of Urological Pathology grade 4. (**B1–B4**) Altered immunoprofiles. The most remarkable findings are aberrant expression of CD10 (**B1**) and alpha-methylacyl-CoA racemase (**B2**) with cytokeratin 7 (**B3**) and c-kit (**B4**) preservation. Scale bars = 1 mm.

ChRCC is primarily characterized by CK7 positivity or being generally diffuse (Figure 6A1,A3) or occasionally patchy (Figure 6A2). CK7-negative tumors are less likely to be ChRCC. EMA is, however, diffusely positive in both tumors (Figure 6B4), whereas CD10 is mostly negative (Figure 6B1), with exceptional focal positivity (Figure 6B2,B3). AMACR is also negative (Figure 6C1) or focally positive (Figure 6C2,C3). E-cadherin is well preserved, never showing loss or broken patterns in both tumors (Figure 6C4). CAIX is completely negative (figure not shown). c-kit labeling is almost always diffuse and strong in both ChRCC (Figure 6D1–D3) and RO (Figure 6D4), but this is not pathognomonic since major parts of renal oncocytoma (RO) are also strongly c-kit-positive. Nearly all ChRCC showed almost total loss of HNF1β as a pathognomonic sign (Figure 6E1,E3) in spite of partial loss (Figure 6E2) as opposed to RO, which showed a totally positive reaction (Figure 6E4). In the present study, ChRCC demonstrated total loss of HNF1β (24/30–80%), but partial loss in 20%, while no mimickers including oncocytoma demonstrated HNF1β loss. In contrast, CK7-/c-kit+/HNF-1β+ staining can suggest oncocytoma. In the case of hybrid tumors with oncocytoma and ChRCC or undetermined cases with overlapping features of ChRCC and renal oncocytoma, triple immunomarkers are very useful. When any tumor demonstrates a CK7 patch positive/c-kit positive/ HNF-1β totally negative result, this favors ChRCC over RO. We optimized the cut-off value of HNF-1β as a positive rate over 50% of total nuclei, since all cases compatible with ChRCC never exceed the positive rate of HNF-1β over 50%. We have another two collections of ChRCC that were proven to be very aggressive and have a fatal course, metastasizing (Figure 7A1) and even causing death within 1 year. Both cases revealed focal spindling features (Figure 7A2) and marked anaplasia (International Society of Urological Pathology nucleolar grade 4) (Figure 7A3). Notably, CD10 (Figure 7B1) and AMACR (Figure 7B2) were aberrantly overexpressed in both aggressive cases, while CK 7 (Figure 7B3) and c-kit (Figure 7B4) maintained expression. 

**Figure 8:**
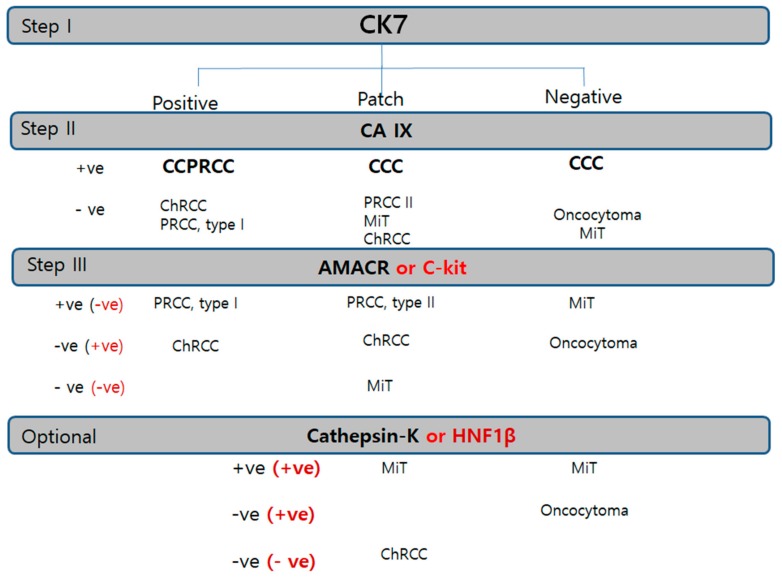
Diagnostic immunohistochemistry algorithm based on the priority or sequence of trial in RCC. Cytokeratin 7 is the first trial universal marker in any kind of renal tumor. Carbonic anhydrase IX is the second trial cardinal marker. Alpha-methylacyl-CoA racemase or c-kit is used as the third panel to be applied in tubular/papillary or solid tumor, respectively. Cathepsin K or hepatocyte nuclear factor-1β is the subsequently used trial for confirmation of MiT family RCC or ChRCC. Abbreviations: CCC, clear-cell carcinoma; ChRCC, chromophobe renal cell carcinoma; CCPRCC, clear-cell papillary renal cell carcinoma; RO, renal oncocytoma; PRCC, papillary renal cell carcinoma; ACD-RCC, acquired cystic disease associated renal cell carcinoma; MiT RCC, microphthalmos gene translocation-associated renal cell carcinoma.

For more efficient and reproducible optimal immunopanels, we proposed two-step panels of a canonical triple panel, CK7/AMACR/CAIX and adjunctive panel in equivocal cases (Figure 8). 

## 3. Discussion 

We suggest a practical immune algorithm to cover almost all renal tumor subtyping. The CK7/AMACR/CAIX triple immunomarker is recommended as the first panel for subtyping of renal epithelial tumors based on the essential speculation in the current survey of immunopanel expression in renal tumors: 1) the importance of CK7 for RCC subtyping as a primary universal cardinal immunomarker, 2) the broad spectrum of AMACR, and 3) the highly specific marker of CAIX. The *CK7−/CAIX++/CD10++* (saw-tooth pattern) panel is a robust immunoprofile for CCRCC. Although light microscopic features suggest CCRCC, when immunohistochemical results of this panel do not follow the above-mentioned profile, diagnosing of CCRCC is very cautious. Instead, we recommend considering other diagnoses of CCRCC mimickers, such as MiT RCC or CCPRCC. In cases of intrarenal tumors which are predominantly composed of clear cells with low grade, the first triple-biomarker panel (CK7-CAIX-AMACR) is sufficient to eliminate the possibility of CCPRCC or several cystic entities. When intrarenal tumors are predominantly composed of mixed clear cells and oxyphilic cells with high grade, we recommend more case-sensitive immunohistochemical panels. Specifically, CAIX++/CK7− results are an error-free immunopanel with prime high fidelity for CCRCC. When the primary panel remains inconclusive, however, parts of subsequent biomarkers (CD10, EMA, cathepsin K, c-kit, HNF1β, and E-cadherin) can be applied. We reconfirmed in the present study that E-cadherin is variably lost in CCRCC [8] and PRCC. CK7 is expressed at low levels in CCRCC, which is the most common clear-cell subtype; unusually high CK7 expression can definitely rule out the possibility of CCRCC, whereas diffuse CK7 positivity is strongly supportive of ChRCC (100%), but less certainty is reported for negative or focal staining [9]. AMACR is a mitochondrial enzyme that mediates fatty-acid oxidation and is commonly expressed in normal hepatocytes, the epithelium of the proximal renal tubules, and the bronchus [10]. Despite its renal origin, it is a very sensitive immunomarker in genitourinary tract cancers, such as renal, bladder, and prostate cancer. AMACR is exclusively useful for both PRCC types I and II in renal tumors. Practically, strong and diffuse AMACR staining is very useful to primarily consider PRCC. A common characteristic of both PRCC types I and II is diffuse and intense expression of AMACR. Recently, type 2 PRCC has been considered a more heterogeneous group than type 1 PRCC [11]. For instance, MiT RCC, ESRC-RCC, CCPRCC overlap papillary patterns. Additionally, fumarate hydratase-deficient RCC (hereditary leiomyomatosis-associated RCC) has been recently documented, which is characteristically composed of large, round cells with prominent nucleoli like Reed–Stenberg cells or viral inclusions [12]. For solid oncocytic tumors with a vaguely papillary pattern, E-cadherin loss with c-kit negativity is useful for differentiating between oncocytic PRCC and ChRCC/oncocytoma. Epithelial markers (CK7, EMA, E-cadherin) are seldom positive in PRCC type II, MiT RCC, and oncocytoma. Merely based on immunoprofiles, PRCC II resembles more closely ACD-RCC than PRCC type I. Other AMACR-positive families include ACD-associated RCC and tubulocystic carcinoma. Lack of AMACR is commonly characteristic of distal nephron-derived renal tumors, such as oncocytoma, ChRCC, or MiT RCC. Since CAIX is regulated by von Hippel–Lindau (vHL) gene products, in CCRCC that frequently lacks vHL, CAIX has been considered the most specific immunomarker [11]. 

Unlike one report about the inverse correlation of CAIX to nucleolar grade, we identified a significant correlation between CAIX and nucleolar grade [12]. The former, however, failed to have statistical significance. Furthermore, since the cut-off criteria were defined as 85%, the low expression in higher grade was unexpectedly high in frequency. When the conventional three-tier algorithm was applied in the present study, the result was shown to be correlative to higher nucleolar grade. 

In many RCC subtypes, CAIX staining inversely correlates with CK7. CCPRCC, which has recently been associated with low-grade malignant behavior [13], manifests characteristic cup-shaped staining with open lumen in contrast to the latter with squared box-shaped pattern [14]. Therefore, both CK7 and CAIX positivity is unique in renal neoplasm of the low-malignant-potential group including multilocular cystic renal neoplasms and in CCPRCC. 

Microphthalmia translocation family (MiT) RCC is one of the most recently documented and underinvestigated subtypes of RCC. Both CK7-CAIX negative results are not so common, only noticed with MiT RCC and renal oncocytoma; fortunately, both are not histologically confusing each other. MiT RCC is a genetic fusion gene-associated malignant tumor caused by uncontrolled overexpression of MiT factors, followed by translocation with several partner genes, such as PRCC-TFE3 [t (X:1) (p11.2; q21.2)], ASPSCR1-TFE3, SFPQ-TFE3, and NONO-TFE3 fusion genes, and so on. Fortunately, all fusion proteins end with TFE3 overexpression, which is diagnostic by virtue of IHC of transcription factors [15]. Ambiguous (patch or weak) TFE3 staining should be considered negative or pending for MiT RCC, but identification of fluorescent in situ hybridization break-apart signals is more convincing [16,17,18]. The molecular approach or specific TFs IHC panels, however, are possible only when considered properly. Moreover, the IHC landscape remains untrustworthy, making it one of the most difficult renal tumors to diagnose. TFEB-related MiT RCC, another emerging TF that has been recently associated with MALAT1-TFEB fusion [t(6;11) (p21.2; q13)], tends to be more melanocytic [19,20,21]. In addition to previously mentioned criteria in the first report as solid nested growth of biphasic tumor cells with basement-membrane materials deposit [19,20,21], we suggest other histologic clues for TFEB-related MiT RCC. When TFE3/TFEB IHC is not available, cathepsin K is a highly sensitive and more broad-spectrum biomarker for MiT RCC. MiT modulates the cathepsin K promotor to control the mRNA and protein expression of papain-like transferase in osteoclasts [16,22,23]. Cathepsin K is very useful to screen for the possibility of MiT RCC, since all intrarenal tumors but MiT RCC are perfectly negative. Foamy histocytes that are seen frequently in PRCC can be used as a positive control of cathepsin K in the initial setting of IHC. The cathepsin K-positive tumor family includes MiT RCC, melanotic Xp11.2 RCC, malignant/borderline epithelioid PEComa, and conventional angiomyolioma [22,24]. Now, we support that MiT RCC is considered to be in the lineage spectrum of PEComa to cause extraordinary coexpression of epithelial and melanocytic markers in renal tumors. When purely melanocytic differentiation occurs with MiT genetic translocation, melanotic MiT tumors may be same as PEComa. Traditional angiomyolipoma (AML) is caused by loss of tuberous sclerosis proteins 1 and/or 2, eventually undergoing mesenchymal differentiation [25]. 

The proto-oncogene *KIT* encodes a transmembrane tyrosine kinase receptor (c-kit/CD117) [26]. Since distal nephrons weakly express c-kit, ChRCC and oncocytoma originating from the distal nephron indicate a positive landmark [27]. In the literature review including our collection, the overall range of frequencies of c-kit expression are 46–100% and 71–100%, respectively [25,26]. However, despite IHC detection for c-kit in most ChRCCs and renal oncocytomas, no activating *KIT* mutations have been demonstrated in renal tumors [28]. In ChRCC, HNF-1β negativity is absolutely unique in ChRCC and its sarcomatoid variants [29,30]. HNF-1β, encoding a member of the homeodomain-containing superfamily of transcription factors, is important in nephron development [31]. The major pathogenesis of sporadic ChRCC is HNF1β inactivation by transcriptional inactivation and post-transcriptional regulation as with tumor-suppressor genes, not by genomic mutations or promotor hypermethylation as in colorectal and ovarian CCRCC [31]. Other important mutations are found in 23.9% of p53 or 10.9% of BHD (Birt–Hoge–Dubé) [27]. Chromophobe RCC is distinctively, in a true sense, an epithelial-friendly tumor in viewpoint of IHC [32,33]. We also suggest that CD10 and AMACR aberrant expression can be a predictor of aggressive behavior in ChRCC in addition to the known factor of sarcomatoid differentiation [34,35]. CD10 has often been documented as a grave prognostic factor in genitourinary tract cancer [35]. Although aberrant cytoplasmic β-catenin is associated with unfavorable RCC [34], unlike one prior study [36], β-catenin proved to be the least supportive marker for subtyping of renal cell tumors in the present study. Immunohistochemical triage in renal tumors according to this algorithm suggested a more convincing and cost-effective efficient algorithm to avoid falling in the trap of unexpected mimickers. 

## 4. Materials and Methods 

### 4.1. Tissue Samples and Data Collection

A total of 208 RCC cases were analyzed using 14 IHC panels of possible candidates to differentiate subtypes. Three major subtypes were CCRCC (50 cases), PRCC (53 cases), which was subdivided into type I (25 cases) and II (25 cases), hereditary leiomatosis RCC (3 cases), and ChRCC (30 cases). Minor subtypes included 20 ACD-RCCs, MiT RCCs (14 cases), comprising 12 TFE3 cases and 2 TFEB cases, 10 CCPRCC cases, 10 multilocular cystic RCCs, 10 oncocytomas, 5 CDC, 3 mixed epithelial and stromal tumors (MEST), 2 tubulocystic RCCs, and one ALK-positive RCC. All patients underwent radical or palliative nephrectomy between March 2010 and January 2015. This study was approved by the Institutional Review Board of Severance Hospital (4-2019-1298). Informed consent was obtained from the patients.

### 4.2. IHC Analysis

After representative areas of CC RCC tissues were identified, IHC analysis was performed on each whole tissue section instead of tissue microarray to avoid intratumoral immunogenic heterogeneity. The primary antibodies are summarized in Table 4. Negative controls were performed by omitting the primary antibody. All were conducted with the Ventana Benchmark XT automated stainer (Ventana Medical Systems, Tucson, AZ, USA) according the manufacturer’s protocol.

### 4.3. Evaluation of IHC Staining

IHC staining was analyzed using a microscope (Olympus, Tokyo, Japan) by three pathologists who were blinded to the disease outcome. For each slide, the three pathologists reached a consensus, and discrepancies were resolved by another consultation. CK7, CAIX, EMA, c-kit, and E-cadherin were valid only when localized along the cytoplasmic membrane, while CD10, AMACR, cathepsin K, and HMB45 were located in the cytoplasm. Immunoexpression was scored by assessing the cytoplasmic staining frequency. Frequency was scaled as 0 (no expression), 1 (1–50%), 2 (over 50%). HNF-1β, TFE3, TFEB, and β -catenin were regarded positive only when nuclei were stained. 

### 4.4. Confirmation of FISH Break-Apart Probe of TFE3 

When TFE3 IHC was equivocal, another validation was confirmed by break-apart FISH. A ZytoLight SPEC *TFE3* Dual-Color Break-Apart Probe (ZytoVision, Bremerhaven, Germany) was used in this study. FISH-positive cases of *TFE3* were defined as samples containing more than 15% split signals in tumor cells. A Zeiss Axio Z2 (Carl Zeiss, Jena, Germany) × 60 objective, and trifilter (40, 6-diamidino-2-phenylindole (DAPI), tetramethyl rhodamine isothiocyanate (TRITC), and fluorescein isothiocyanate (FITC)) were used to score each case.

## 5. Conclusions

Increasingly tremendous subtypes of renal epithelial tumors need more comprehensive and accurate IHC performance, in association with appropriate selection of antibodies and each targeted interpretation. An adjunctive panel can be suggested, including CD10, EMA, cathepsin K, c-kit, HNF1β, and E-cadherin, following a primary screening panel of the CK7-CAIX-AMACR algorithm, which is the most efficient strategy for subtyping of RCC. 

## Figures and Tables

**Table 1:** Differential immunohistochemistry panels in cystic renal tumors.

Marker	Multilocular Cystic RCC (n = 10)	Cystic CCPRCC (n = 10)	ACD-RCC (n = 20)	Tubulocystic Carcinoma (n = 2)
CK7	10/10 (100)	10/10 (100)	5/20 (25)	0/2
AMACR	3/10 (30)	1/10 (10)	12/20 (60)	2/2 (100)
CAIX	10/10 (box shape)	10/10 (cup shape)	0/20	0/2
CD10	8/10 (80)	8/10 (80)	20/20 (100)	0/2
EMA	8/10 (80)	9/10 (90)	15/20 (75)	0/2
E-cadherin	5/10 (broken)	10/10 (100)	14/20 (broken) (70)	0/2

Abbreviations: RCC, renal cell carcinoma; CCPRCC, clear-cell papillary RCC; ACD-RCC, acquired cystic disease associated renal cell carcinoma; CK7, cytokeratin 7; AMACR, alpha-methylacyl-CoA racemase; CAIX, carbonic anhydrase IX; EMA, epithelial membrane antigen.

**Table 2:** Triple immunomarkers as the first trial panel in RCC subtyping.

Tumor Subtype	CK7			CAIX			AMACR		
	0	1+	2+	0	1+	2+	0	1+	2+
CCRCC (n = 50)	10	90	0	0	0	100	4	70	26
PRCC I (n = 15)	0	6	94	100	0	0	0	7	93
PRCC II (n = 35)	83	17	0	9	77	14	0	11	89
ChRCC (n = 20)	0	15	85	100	0	0	65	35	0
RO (n = 10)	80	20	0	100	0	0	100	0	0
CCPRCC (n = 10)	0	0	100	0	0	100	80	20	0
MiT RCC (n = 13)	100	0	0	100	0	0	62	38	0
CDC (n = 5)	0	0	100	100	0	0	0	40	60

Negative: 0, focally positive: 1+, diffusely positive: 2+. Each number represents the precentage of the expression of markers. Abbreviations: CK7, cytokeratin 7; AMACR, alpha-methylacyl-CoA racemase; CAIX, carbonic anhydrase IX; CCRCC, clear-cell carcinoma; PRCC, papillary renal cell carcinoma; ChRCC, chromophobe renal cell carcinoma; RO, renal oncocytoma; CCPRCC, clear-cell papillary renal cell carcinoma; MiT RCC: microphthalmos gene translocation-associated renal cell carcinoma; CDC, collecting duct carcinoma.

**Table 3:** Additional hexapanels for adjunctive renal cell carcinoma subtyping.

Subtype	CD10	EMA	E-Cadherin	c-Kit	HNF-1β	Cathepsin K
CCRCC	2+ (saw-tooth)	1+	1+ (broken)	0	2+	0
PRCC I	0	0	0	0	2+	0
PRCC II	2+(tram track)	2+	0	0	2+	0
CCPRCC	1+	1+	1+ (broken)	0	2+	0
ChRCC	0	2+(cytoplasm)	2+	2+	0–1+	0
RO	0	2+(cytoplasm)	2+	1+	2+	0
MiT RCC	0	0	1+	0	2+	2+
CDC	0	1+(cytoplasm)	0–1+ (loss-broken)	0	2+	0

Abbreviations: EMA, epithelial membrane antigen; HNF-1β, hepatocyte nuclear factor-1β; CCRCC, clear-cell renal cell carcinoma; PRCC, papillary renal cell carcinoma; ChRCC, chromophobe renal cell carcinoma; CCPRCC, clear-cell papillary renal cell carcinoma; RO, renal oncocytoma; MiT RCC: microphthalmos gene translocation-associated renal cell carcinoma; CDC, collecting duct carcinoma.

**Table 4:** Information on primary antibodies used for immunohistochemical analysis.

Antibody	Catalog No.	Company	Nation	Dilution Factor
CD10	L-CD10-270	NOVOCASTRA	UK	100
CK7	M7018	DAKO	Denmark	100
c-kit	A4502	DAKO	Denmark	1000
Cathepsin K	ab37259	Abcam	UK	400
CAIX	NB100-417	Novus Biologics	USA	1000
P504 (AMACR)	M3616	Dako	Denmark	100
EMA	M0613	DAKO	Denmark	200
E-cadherin	M3612	DAKO	Denmark	100
HMB45	M0634	DAKO	Denmark	300
β-catenin	224M-15	Cell Marque	USA	200
TFE3	354R-14	Cell Marque	USA	100
TFEB	ab2636	Abcam	UK	400

Abbreviations: CK7, cytokeratin 7; CAIX, carbonic anhydrase IX; AMACR, alpha-methylacyl-CoA racemase; EMA, epithelial membrane antigen; TFE3, transcription factor E3; TFEB, transcription factor EB.

## Supplementary Materials

The following are available online at https://www.mdpi.com/2072-6694/12/3/602/s1, Figure S1: Fumarate hydratase deficiency-associated RCC.
