# Supplementary material for: Comprehensive Immunoprofiles of Renal Cell Carcinoma Subtypes
Source: Cancers (Basel). 2020 Mar 5;12(3):602. doi: 10.3390/cancers12030602 (PMC7139472; doi:10.3390/cancers12030602)
Supplement: Supplementary file 1 [file cancers-12-00602-s001.pdf]

**Supplementary Materilas**

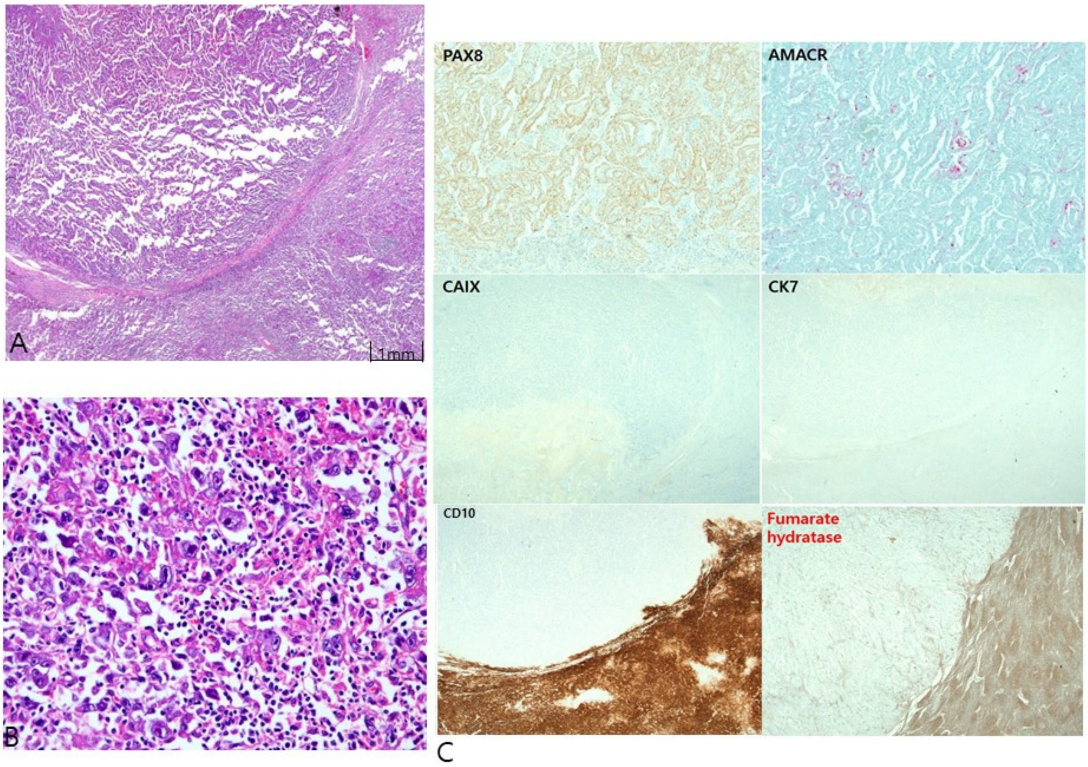


**Figure S1.** Fumarate hydratase-deficiency associated RCC. A: Multinodular mass showing papillary and solid forming growth pattern, easily categorized to papillary type II with sarcomatoid features. B: Pleomorphic tumor cells with prominent meganucleoli mimicking viral inclusion or Reed-Stenberg cell. C. Immunohistochemical panels are displayed. PAX8 is positive, but conventional markers including CK7/AMACR/CAIX/CD10 are all negative. Total loss of FH is a specific clur to FH-deficient RCC. Scale bars = 1 mm.
